# Heterochronic genes in plant evolution and development

**DOI:** 10.3389/fpls.2013.00381

**Published:** 2013-09-25

**Authors:** Koen Geuten, Heleen Coenen

**Affiliations:** Department of Biology, Laboratory of Molecular Plant Biology, University of LeuvenLeuven, Belgium

**Keywords:** microRNA172, microRNA156, *choripetala*, *incomposita*, *Ceratopteris*, heterochrony, APETALA2, SPL

## Abstract

Evolution of morphology includes evolutionary shifts of developmental processes in space or in time. Heterochronic evolution is defined as a temporal shift. The concept of heterochrony has been very rewarding to investigators of both animal and plant developmental evolution, because it has strong explanatory power when trying to understand morphological diversity. While for animals, extensive literature on heterochrony developed along with the field of evolution of development, in plants the concept has been applied less often and is less elaborately developed. Yet novel genetic findings highlight heterochrony as a developmental and evolutionary process in plants. Similar to what has been found for the worm *Caenorhabditis*, a heterochronic gene pathway controlling developmental timing has been elucidated in flowering plants. Two antagonistic microRNA’s miR156 and miR172 target two gene families of transcription factors, SQUAMOSA PROMOTOR BINDING PROTEIN-LIKE and APETALA2-like, respectively. Here, we propose that this finding now allows the molecular investigation of cases of heterochronic evolution in plants. We illustrate this point by examining microRNA expression patterns in the *Antirrhinum majus incomposita *and* choripetala *heterochronic mutants. Some of the more beautiful putative cases of heterochronic evolution can be found outside flowering plants, but little is known about the extent of conservation of this flowering plant pathway in other land plants. We show that the expression of an *APETALA2-like* gene decreases with age in a fern species. This contributes to the idea that ferns share some heterochronic gene functions with flowering plants.

## INTRODUCTION

Time is a fundamental aspect of all developmental processes. It plays a role in different types of development, such as growth or differentiation and at different scales, whether it be cellular, at the tissue or at the organ level (Moss, 2007). In the discipline of evolution and development, evolutionary changes in the regulation of developmental time or “heterochrony” were once proposed to explain much of morphological diversity (Gould, 1977). To test whether this is indeed the case, it is necessary to be able to unambiguously identify cases of heterochrony. Developmental time is now known in several organisms to be controlled by endogenous mechanisms that interact with endogenous and environmental stimuli (Slack and Ruvkun, 1997, 1998; Moss, 2007; Huijser and Schmid, 2011). Also in plants, a “heterochronic pathway” has been elucidated (Chuck et al., 2007a, b; Wu et al., 2009). For both plants and animals, several classic examples of morphological evolution have been proposed to be heterochronic in nature, suggesting that the regulation of a heterochronic pathway has evolved in these instances. The discovery of a heterochronic pathway contributes to the testability of these hypotheses of regulatory evolution. Ultimately, it should become possible to answer such questions as, “Is evolution of developmental timing frequent in plants?,” “Can it explain most of morphological diversity?,” “What types of morphological consequences can evolution of developmental timing have?” Or more generally, is heterochrony indeed such an important developmental process in the evolution of morphology? Here we mostly review some of the elaborate literature on heterochronic evolution and how it can be applied in the field of plant evolution and development.

## TIME AND RATE IN PLANT DEVELOPMENT

**FIGURE 1:**
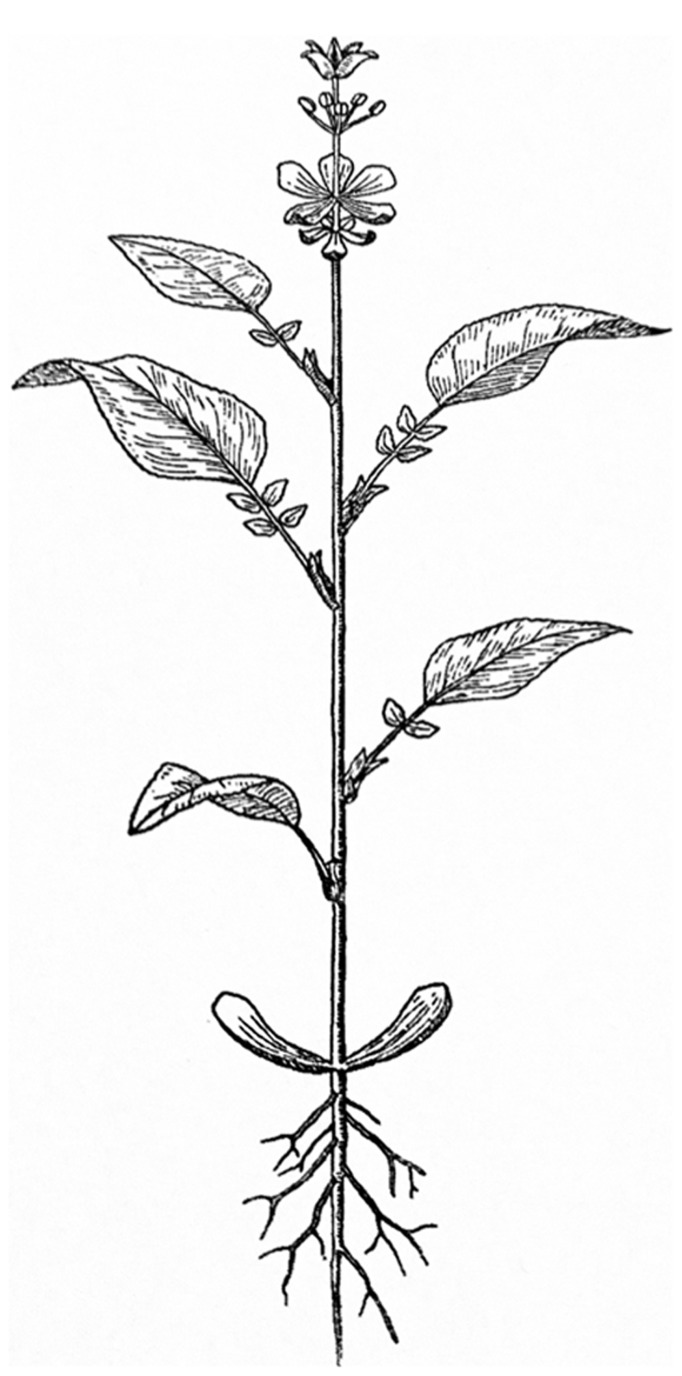
**The annual plant as viewed by Goethe (represented by Troll)**.

In contrast to animal development, plant development entails the continuous development of new organs as time progresses. This open developmental shoot system generates different organs depending on the age of the plant. The different types of above ground leaf-like organs that develop result in a “heteroblastic” sequence observable in the mature plant (Allsopp, 1967; Zotz et al., 2011). This sequence starts with embryonic leaves or cotyledons, then juvenile leaves develop, these transition into adult leaves and finally inflorescence leaves or bracts develop. The floral organs can be seen as a continuation of this sequence of different leaf types, with sepals and petals still resembling leaves. While stamens and carpels do not resemble leaves in most species, they can still be interpreted as such (**Figure 1**; von Goethe, 1790; Arber, 1937; Takhtajan, 1976). A similar sequence can be observed in monoecious inflorescences, in which lower positioned unisexual male flowers develop earlier than unisexual female flowers. Also in cleistogamous species, the different floral types are associated with a timed change in identity resulting in either closed flowers that obligately self or open flowers that can outcross (Lord and Hill, 1987).

Regular time obviously progresses at a constant rate. However, what is called developmental time or age can be fast or slow, meaning that development can be accelerated or retarded relative to regular time or relative to other developmental events (Poethig, 2003). Developmental time or, e.g., plastochron length in case of leaves, is the time that passes between the development of two successive leaves. Developmental rate in plants is counted in numbers of organs that develop per unit of time. Plants are special in this sense because developmental rate can be easily measured in the adult form of the plant as an average number of organs that has developed in a certain period of time, which makes plants a good system to study developmental time.

## DEFINITIONS OF HETEROCHRONY

The term heterochrony was first introduced by Ernst Haeckel in the second half of the 19th century (Smith, 2003). It was used to describe deviations from his well-known “Biogenetic Law” which states that the sequence of developmental events largely recapitulates the sequence of events in the evolutionary history of the species. In several books, de Beer uncoupled heterochrony from recapitulation and used the term to denote a relative displacement of a character in its timing of development when comparing two related species (De Beer, 1951). Gould (1977), in his reevaluation of the concept, focused heterochrony again on parallels between or reverse relations of ontogeny and phylogeny and emphasized size and shape as the measures to detect heterochrony. The way the concept has most often been applied and tested is therefore through morphological measurements. Because in development, size and shape tend to change through growth, cases of heterochrony have been documented through a quantitative analysis of size and shape, called allometry (Gould, 1977; Klingenberg, 1998; Webster and Zelditch, 2005; Box and Glover, 2010). This resulted in detailed descriptions of quantitative variation of morphology and inferences of heterochrony. Heterochrony is not limited to morphological observations though and a developmental viewpoint of the concept was elaborated in Raff and Wray (1989). More recently, Smith (2001) untangled the different historical meanings of the term heterochrony by recognizing two identifiable types: “growth heterochrony,” following Gould, and “sequence heterochrony,” more in line with the original usage of Haeckel and de Beer that focuses on the relative timing of developmental events. Here we wish to mainly consider the heterochronic morphological consequences of certain developmental control genes that can also be viewed as heterochronic. Such a direct link between a heterochronic underlying mechanistic process and a morphological result can, in our view, contribute to the testability of putative morphological cases of heterochrony in either mutants or evolutionary examples. Therefore, we aim to provide the term heterochrony with a clear molecular basis, without aiming to limit or redefine its meaning.

## THE RELATION OF HETEROCHRONY TO OTHER MODIFICATIONS OF DEVELOPMENT

At first, it appears easy to contrast heterochrony to other modifications that can occur in the evolution of development. Heterotopy for instance, is defined as a developmental process affected in location, while heterochrony is a process affected in time. However, a shift in timing of development can also result in a change in location. For instance a petal primordium could develop later than usual and as a consequence also shift in position. This illustrates that strictly using morphological observations, it is difficult to distinguish modifications in ontogeny. In previous discussions of heterochrony in plants, there was no mention yet of heterochronic genes or a pathway identified. However, now that a pathway has been identified, by investigating the mechanistic (molecular) process behind a morphological change, a distinction could be made between heterochrony and heterotopy based on the underlying genes affected. A further problem is how to distinguish heterochrony and heterotopy from homeosis, another important category in evolution of development which entails the transformation in evolution of the identity of an organ. We would argue that homeosis can be the result of both heterochrony and heterotopy. For instance a sepal to petal transformation can result from a spatial shift of the petal identity program, but it could also result from a heterochronic shift.

One explicit criticism is that heterochrony is unable to explain the origin of new structures in evolution, as only a shift in time of an existing process is meant by the term (Horder, 2013). However, the same criticism could be voiced against homeosis and heterotopy and relates more to the effect of the shift being dynamic or static (Webster and Zelditch, 2005). When dynamic, a modification in size, shape, or identity of the structure occurs during the shift, while when static the structure is only repositioned in time or location.

## TYPES OF HETEROCHRONY

**FIGURE 2:**
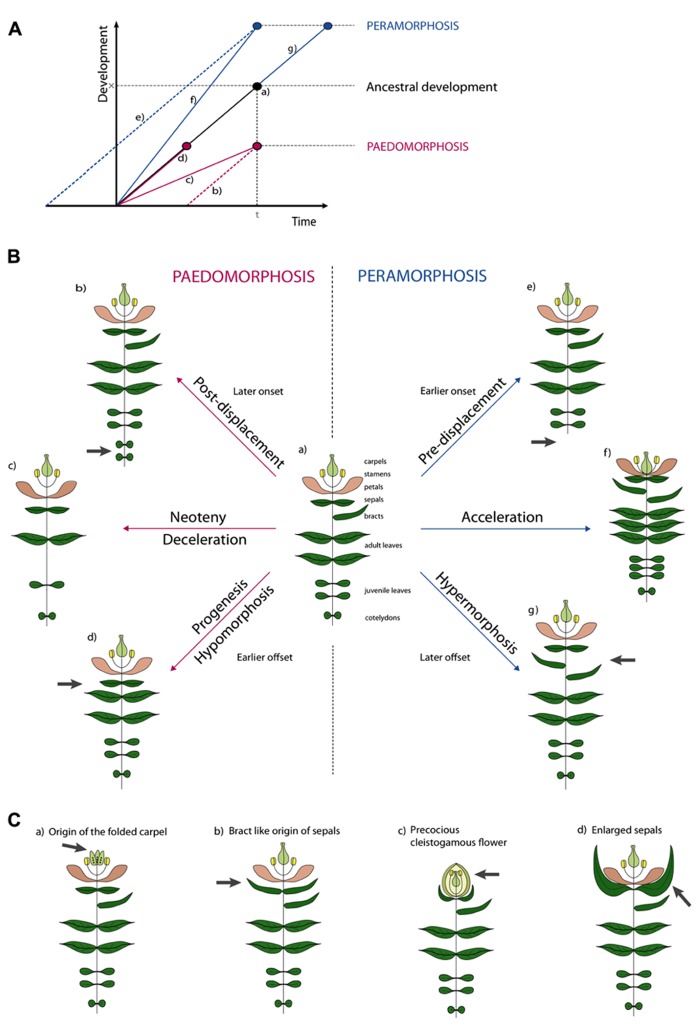
**Illustrative examples of types of heterochronic evolution. (A)** Schematic overview of the different types of heterochrony. Developmental time is the time span required to reach a certain developmental stage. In normal ancestral development **(a)** an organism requires time span *t* to reach development stage *x*. In Paedomorphosis development is reduced. Depending on the cause three subtypes can be distinguished. Delayed onset is the cause in post-displacement **(b)**. In neoteny **(c)** development rate is slowed down. In progenesis **(d)** the normal time span is shortened, development will stop prematurely. In Peramorphosis an extended level of development is achieved. Again, we distinguish three subtypes. In pre-displacement **(e)** an earlier onset will result in a prolonged time span of development or in early maturation. In acceleration **(f)** developmental rate is increased. Finally, in hypermorphosis **(g)** development is continued after normal offset. **(B)** Hypothetical examples of heterochrony. Figure **(a)** shows a reference plant with different types of plant organs. Peramorphic develop “beyond” this, while paedomorphic plants retain juvenile features. In post-displacement **(b)**, onset is later and an additional pair of cotyledons develops, in pre-displacement **(e)**, onset is later and no cotyledons develop. In neoteny, less organs develop with larger internodes **(c)**, while in acceleration **(f)**, more organs develop with shorter internodes. In progenesis **(d)**, offset is earlier and no bract develop, while in hypermorphosis **(g)**, offset is later and additional bracts develop. **(C)** Examples of heterochrony taken from the literature. See text for explanation.

Several attempts have been made to classify types of morphological heterochrony based on the possible outcomes of allometric studies and the two major types have been termed paedomorphosis and peramorphosis. Paedomorphosis results in juvenile structures in adult stages while peramorphosis results in the extended development of structures. Either form can be explained by three developmental causes, as proposed in the typology of Alberch et al. (1979). The causal subdivisions rely on the analogy of development being linear (**Figure 2A**). This “developmental line” is determined by (1) a point of onset or the start of development, (2) a rate of development represented by the local slope of the line, and (3) an offset of development (McNamara, 2012).

We illustrate this typology for simple and hypothetical cases of heterochrony in plants as shown in **Figure 2B**. While depicting development would in principle require several growth stages for every example, for plants it suffices to draw only the adult stage because these stages are retained in the adult plant (**Figure 2B**). When underdevelopment in paedomorphosis is caused by a delay in onset, this is called post-displacement (**Figure 2B, b**). So far no such case has been described in plants (Li and Johnston, 2000), but a hypothetical example could be the development of more cotyledons, with a delay of the onset of the juvenile phase as a result. The term neoteny is used when paedomorphosis is the result of a slower developmental rate. For example, fewer leaves could develop as illustrated in (**Figure 2B, c**). As an extreme example, the entire adult plant could retain juvenile characters, such as in species of Lemnaceae. A third paedomorphic subtype, progenesis, is used when offset occurs earlier. One example could be the omission of a terminal developmental stage like bract development in **Figure 2B, d**. Contrary to paedomorphosis, characters develop “beyond” the ancestor in peramorphosis. When onset of developmental processes is earlier, we use the term pre-displacement (**Figure 2B, e**). For example, juvenile leaves develop immediately, without developing cotyledons first. When developmental rate is faster we use acceleration (**Figure 2B, f**). A result of an acceleration could be the development of more organs and finally the contraction of internodes between floral organs in the flower. Finally in hypermorphosis, development beyond the offset point, can result in the development of additional organs, such as bracts (**Figure 2B, g**).

It becomes more difficult when one wants to apply this terminology to more realistic examples (**Figure 2C**). For instance, neoteny according to Takhtajan (1976) could explain the origin of the folded carpel (**Figure 2C, a**). Ancestral carpels would origin from unfolded leaf-like structures which had to pass through a juvenile stage of unfolding. The derived folded carpels thus resemble the juvenile folded stage. But also the origin of sepals from bracts has been hypothesized to be neotenic (Arber, 1937; Takhtajan, 1976). Sepals can be interpreted as small leaves and seem juvenile in a way (**Figure 2C, b**). Alternatively, we think the latter could be named progenesis, because development of bracts stops earlier with a smaller leaf as a result. Cleistogamous flowers have been a classic example of heterochrony (**Figure 2C, c**). Where Gould (1988) used to believe that the cleistogamous flower was a paedomorphic “progenetic dwarf” version of the chasmogamous flower (progenesis), several studies showed that different heterochronic processes are involved in the resulting precocious, but unopened and smaller, flower (Mayers and Lord, 1983a, b; Li and Johnston, 2000; Porras and Muñoz, 2000). In cleistogamous flowers of *Viola odorata* for example, pollen maturation initiates earlier compared to the ancestral chasmogamous flower (pre-displacement). But not only the early onset of meiotic processes will lead to precocious flowers, an increased leaf initiation rate and flower formation (acceleration) and a repressed cell expansion (progenesis) will contribute to the final phenotype. Finally, enlarged sepals have been interpreted as vegetative characters displaced into reproductive development (**Figure 2C, d**). A good example is the inflated calyx of *Physalis* species after fertilization. This could be interpreted as hypermorphosis, as the organ develops beyond its normal growth and the vegetative character extends into the reproductive phase.

From these examples it becomes clear that identifying the exact type of heterochronic evolution in more realistic examples is often difficult and it can be expected that more than one type is involved in many cases of heterochronic evolution (Li and Johnston, 2000). The identified type of heterochrony can also depend on the chosen point of reference. In animals, the most often chosen point of reference is sexual maturity. In plants, several other points of reference have been used like the initiation of primordia as an onset, or anthesis as offset (Li and Johnston, 2000; Box and Glover, 2010).

## A HETEROCHRONIC PATHWAY IN FLOWERING PLANTS

While extensive literature is available on heterochrony, its definition and typology, at least for plants this does not take into account a now known mechanistic basis of developmental timing. In this paragraph, we provide an updated brief introduction to a basic mechanism of developmental timing, which is extensively reviewed elsewhere (Chuck et al., 2009; Huijser and Schmid, 2011; Zhu and Helliwell, 2011; Schwab, 2012; Yamaguchi and Abe, 2012). What we denote here as one recently discovered “heterochronic pathway” is more specifically the sequential and antagonistic function of two microRNA’s, their upstream regulators and downstream effectors or targets.

It has been established that two microRNA’s, *miR156* and *miR172*, act as the main players in the regulation of developmental timing in flowering plants (Chuck et al., 2007a, b; Wang et al., 2009; Wu et al., 2009). In early stages of development, *miR156* levels are high and they decrease during plant development, while *miR172* shows the opposite pattern. These microRNA’s contribute to both the juvenile-adult phase transition and the transition to flowering through their sequential and antagonistic actions (Wu et al., 2009). *miR156* represses targets of the *SQUAMOSA PROMOTOR BINDING PROTEIN-LIKE* (*SPL*) gene family and maintains juvenile features of the plant (Schwab et al., 2005). When *miR156* levels decline, the SPL proteins increase and will activate *miR172*, activate flowering genes, and induce adult leaf features (the functional evolution of SPL genes is reviewed in Preston and Hileman, 2013). *miR172* targets 6 members of the AP2-like transcription factor family in *Arabidopsis* (Aukerman and Sakai, 2003). During the stages when *miR172* levels are increasing, the *APETALA2-like* (*AP2-like*) genes are progressively silenced and adult leaf traits and flowering is induced.

Even though much progress is made in understanding the regulation of phase transitions through microRNA’s and their downstream effectors, the upstream molecular mechanisms are just starting to be understood only in *Arabidopsis thaliana*. Recently it was shown that sugars control the *miR156* age-dependent decrease (Proveniers, 2013; Yang et al., 2013; Yu et al., 2013). When growing older, the plant accumulates sugar through increasing photosynthesis activity. Sugar in turn represses *miR156* expression at the transcriptional and post-transcriptional level, causing *miR156* to decrease (Yang et al., 2013; Yu et al., 2013). *miR172* levels can also be influenced by other environmental factors. SHORT VEGETATIVE PHASE (SVP) binds directly to the *pri-miR172a* promoter and represses transcription at low ambient temperatures (Cho et al., 2012a). FCA (a RNA binding protein) on the other hand stimulates *pri-miR172* processing at high ambient temperatures (Jung et al., 2012). Both genes are thus involved in the ambient temperature-dependent regulation of *miR172* and will delay flowering when ambient temperatures are low. These mechanisms correspond to the increase in *miR172* abundance at 23°C compared to 16°C (Lee et al., 2010). In contrast, *miR156* is upregulated at 16°C compared to 23°C, but the molecular mechanism causing this has not been identified (Lee et al., 2010). Photoperiod, and more precisely long days, seems to upregulate *miR172* levels as well. In *gigantea* (*gi*) mutants *miR172* was decreased, while there was no effect in *constans* mutants. GI probably executes this function by affecting *miR172* maturation rather than transcription (Jung et al., 2007). Other environmental factors like salinity or UV-stresses and phosphate starvation can cause similar changes in mature *miR156* and *miR172* levels, but underlying mechanisms remain unresolved (Zhou et al., 2007; Hsieh et al., 2009; Gu et al., 2010).

Developmental timing has been extensively studied from the viewpoint of the juvenile–adult transition and the transition to flowering. Yet, *miR172* and its target AP2 have important regulatory roles in flower development as well. First of all a negative feedback regulation exists between *miR172* and AP2 in the flower to help establish floral organ identity (Grigorova et al., 2011; Wollmann et al., 2010). In the two inner whorls *miR172* represses AP2 to guarantee stamen and carpel development (Aukerman and Sakai, 2003; Chen, 2004), while in the two outer whorls AP2 together with co-repressors SEUSS and LEUNIG represses *miR172* by binding directly to the *miR172* promoter sequence in order to develop sepals and petals (Grigorova et al., 2011). In addition AP2, together with TOPLESS and HDA19, also functions as a repressor in the regulation of the outer boundaries of expression of organ identity genes belonging to B, C, and E classes (Krogan et al., 2012). Finally, in order to establish floral determinacy, *miR172* and AGAMOUS cooperate to downregulate *WUSCHEL* expression which is enhanced by AP2. A recently discovered player, *POWERDRESS*, will promote transcription of *miR172a*, *b,* and *c* genes in order to confer this floral determinacy by impeding AP2 accumulation (Yumul et al., 2013).

In conclusion we can say that aside from the well known downstream targets, more and more upstream regulators are being identified. All these genes can thus influence the heterochronic pathway by regulating the microRNA expression levels and can be considered heterochronic genes.

## ORIGIN AND EVOLUTION OF HETEROCHRONIC GENE FUNCTIONS IN LAND PLANTS

The pathway described above has been best studied in *Arabidopsis thaliana* and maize, and its basic function in controlling developmental timing is likely to be conserved in flowering plants, gymnosperms, and to some extent in ferns (Axtell and Bartel, 2005; Shigyo et al., 2006; Axtell et al., 2007; Floyd and Bowman, 2007; Huijser and Schmid, 2011). In addition the interaction of miR156 with its targets is probably also conserved in mosses (Arazi et al., 2005; Axtell et al., 2007). However, in the moss model system *Physcomitrella patens,* *miR156* promotes the development of leafy gametophores, suggesting that its function in flowering plant sporophytes evolved from an opposite function in moss gametophytes (Cho et al., 2012b). Much about its functional origins in sporophyte development may be learned from studying this microRNA in lycopods, ferns, and gymnosperms. Yet precisely for these plant lineages researchers are confronted with strong methodological limitations, such as the inability to genetically modify species.

The interaction of miR172 and its targets probably originated after the divergence of mosses, lycopods (Floyd and Bowman, 2007), and probably ferns. MIR172 like sequences may be present in the moss *Physcomitrella* genome and the water fern *Marsilea *(unpublished genbank data) as evident from bioinformatic sequence analysis, but their expression levels are extremely low or absent, suggesting that these sequences are not functional in mosses and probably in ferns (Fattash et al., 2007; Axtell and Bowman, 2008). The biological significance of these sequences therefore remains unclear. Also our own cloning efforts did not result in miR172 sequences from selected fern species. This is in contrast to the miR172 binding site in an *APETALA2-like* putative target which is present in ferns but not in lycopods. In the *AP2-like* sequences of the lycopod *Selaginella,* no miR172 binding site is present (Floyd and Bowman, 2007).

**FIGURE 3:**
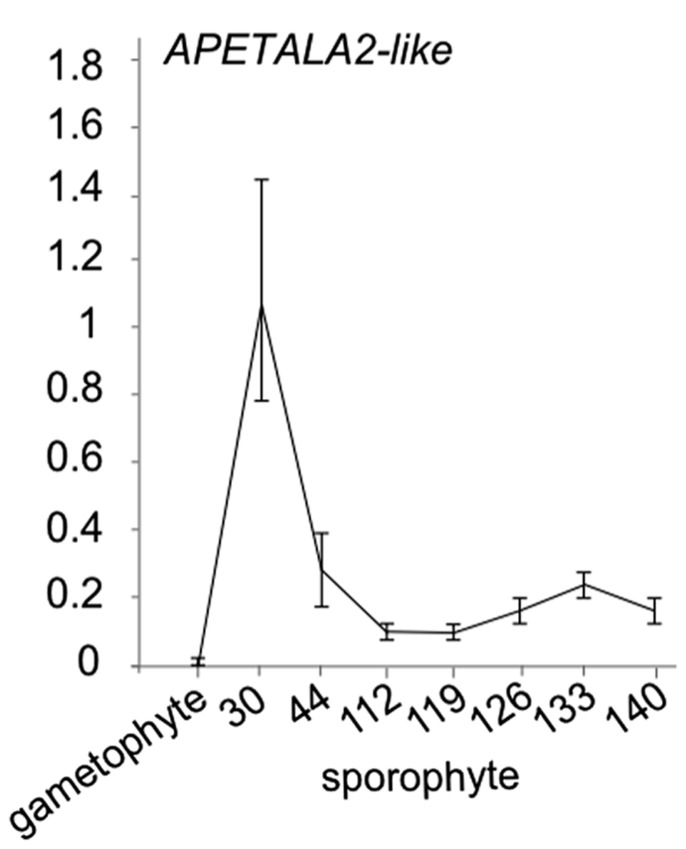
**APETALA2-like expression in *Ceratopteris richardii* developmental time series.** qRT-PCR of a putative miR172 target *APETALA2-like* gene with a putative *miR172* binding site in gametophytes and sporophyte stages of development. Error bars are standard deviation of three biological replicates.**

While little evidence is available for the presence of miR172 in ferns, in *Ceratopteris thalictroides, *a putative miR172**target sequence has been cloned (Axtell and Bartel, 2005). In the absence of a convincing sequence or expression pattern for a mature miR172 microRNA in ferns, an open question is whether the cloned *APETALA2-like* genes with miR172 binding site is progressively downregulated in *Ceratopteris* development. To investigate whether this *APETALA2-like* putative target displays an expression decrease during sporophyte development in ferns, we investigated its expression using qRT-PCR in a developmental time series of the fern *Ceratopteris richardii *sporophyte (**Figure 3**). We indeed observed a decrease in expression levels with an increased developmental age. Interestingly, we did not find expression to be detectable in the *Ceratopteris* gametophyte, suggesting that this target gene only functions in sporophyte development. These data add to the idea that developmental timing is regulated by *AP2-like* genes in ferns. The data together suggest that the binding site in *AP2* likely evolved before the origin of the cognate microRNA, but that *AP2-like *genes already are involved in developmental timing in ferns. Because of the likely absence of *miR172* in ferns, an alternative mechanism may be responsible for the progressive down regulation of *AP2*.

## TIME-DEPENDENT GRADED EXPRESSION OF HETEROCHRONIC GENES AND PLANT ORGAN IDENTITY

A central idea behind heterochrony in plants is that a graded signal exists that changes with time and establishes the identity of the plant organs. In vegetative development this graded signal has been shown for different types of leaves and is referred to as heteroblasty (Allsopp, 1967; Kerstetter and Poethig, 1998; Poethig, 2010). In maize, *Arabidopsis* and poplar, it has been shown that transformation of leaf type can be established by manipulating expression levels of microRNA156 (Wu and Poethig, 2006; Chuck et al., 2007a; Wu et al., 2009; Wang et al., 2011). This suggests that the relative expression level of microRNA’s determine the age of the shoot apex that develops leaf primordia (Schwab, 2012). In the only molecular study that includes multiple species, Wang et al. (2011) also show that juvenile and adult leaf type correlate with these expression levels in *Hedera*, *Acacia,* and *Quercus*, which are classic examples of heteroblasty.

Even von Goethe already noted not only that the different vegetative plant organs appeared remarkably similar to each-other, as if they metamorphosed from one type into another. He also applied the same idea to the reproductive organs (**Figure 1**). Only *miR172* levels have been analyzed in detail during flower development of *Arabidopsis thaliana *(Wollmann et al., 2010). Throughout flower development they showed a graded expression pattern from the outer whorls to the inner whorls. *miR172* expression was higher in the shoot apical meristem than in early flower primordia and in successive stages of flower development *miR172* became increasingly restricted to the center. In the latest stages expression was only detected in developing ovules. This suggests that indeed, also for floral organs a different relative expression level of at least *miR172* characterizes the floral organs. If indeed, *miR172* is heterochronically regulating organ identity in the flower, we would expect that partial knock-down would result in petals transforming into sepals, stamens into petals, and carpels into stamens. Complementary, constitutive expression would result in upward identity shifts along the floral axis. Partially consistent with this prediction, knock-down of *miR172* in stamens results in a partial transformation to petals in *Arabidopsis* (Wollmann et al., 2010). A problem with *miR172* knock-down is that one is not necessarily able to generate slightly different levels that result in predictable transformations (Todesco et al., 2010). For this, the genetic analysis of the different *miR172* genes will be illuminating. While *miR172* ectopic expression in *Nicotiana benthamiana* results in the transformation of sepals into petals (Mlotshwa et al., 2006), *miR172* ectopic expression in *Arabidopsis* results in sepals transformed into carpels and the absence of petals, strongly resembling the *ap2* mutant (Chen, 2004). It could be that in *Arabidopsis*, constitutive expression under the 35S promoter attains too strong level to obtain the expected series of organ identity transformations dependent on the expression level of *miR172*. Consistent with this idea is that ectopic ovules also develop on the leaves in these plants and entire gynoecia in the axil of leaves (Aukerman and Sakai, 2003).

## A HETEROCHRONIC INTERPRETATION OF APETALA2 GENE FUNCTIONS

As *APETALA2-like* genes are under the direct control of miR172, their functions can also be interpreted as heterochronic. While originally, *Arabidopsis* *APETALA2* function was interpreted as A-function in the ABC model (Coen and Meyerowitz, 1991), multiple functions for *APETALA2-like* genes can now be distinguished. Two functions are involved in the timing of identity transitions, either from the shoot apical meristem into a flower meristem or in the identity transitions of floral organ primordia. A third function is in floral determinacy, the end or offset of development.

A first function of *APETALA2* is in timing the specification of the floral meristem by repressing vegetative characteristics from flowers. This function is clear from the phenotypes in several species in which flowers acquire vegetative characters such as the development of bracts and supernumerary sepals (Litt, 2007). This is the case for *Arabidopsis* *ap2* alleles, in which sepals are often transformed into bracts or leaf-like structures (Bowman et al., 1989). Similar phenotypes have been observed for *Antirrhinum* *lip1/lip2* mutants and also in the rice homolog *supernumerary bracts* and the maize double mutant* indeterminate spikelet 1/sister of indeterminate spikelet 1* (Keck et al., 2003; Lee et al., 2007; Chuck et al., 2008). The heterochronic interpretation of these phenotypes is that because of a delayed transition from inflorescence meristem to floral meristem supernumerary bract-like organs develop.

A second function is in timing the identity of the floral organs. In the ABC model, it was proposed that a floral A-function exists that acts to repress C-function from the outer floral organ whorls and contributes to the establishment of sepal and petal identity. Recent findings in *Arabidopsis* show that the repressive function of *APETALA2 *is more general and that the outer boundaries of B-function (APETALA3 and PISTILLATA), E-function (SEPALLATA3), and C-function are marked by APETALA2 (Krogan et al., 2012). The classic ABC model with homeotic functions may thus alternatively be viewed as a combination of heterochronic and heterotopic functions to specify floral organ identity. Heterotopic functions would involve only B- and C-function added onto a ground state of floral meristem identity (Litt, 2007).

*APETALA2* mutants in different species also show other heterochronic phenotypes. A mutant with a weaker phenotype in a maize *AP2-like* gene is *glossy15*, which develops adult characteristics in juvenile leaves (Lauter et al., 2005). In barley, the *cleistogamy1 *mutant was positionally cloned and identified as an *AP2-like *gene (Nair et al., 2010) which is interesting considering that cleistogamous flowers are a classic example of heterochrony (Lord and Hill, 1987).

## DETECTING HETEROCHRONY THROUGH HETEROCHRONIC PATHWAY GENES

Previous review literature on heterochrony in plants (Lord and Hill, 1987; Li and Johnston, 2000; Box and Glover, 2010), discusses the concept of heterochrony in terms of morphological changes in development and not in terms of heterochronic genes. A problem with a morphological definition of heterochrony is that it can become too broadly applicable. Any type of growth or induction at every scale has a time aspect attached to it and such heterochronies would likely have many different underlying causes. This would contribute little to the use of the term heterochrony. Examples of proposed heterochronic evolution may then seem naive or the application of the term does not appear useful anymore. For instance, it is possible to use the term progenesis for a population of *Arabidopsis thaliana*, either mutant or natural, that flowers early. The question is whether applying such a term contributes much to our understanding of evolution. It soon becomes possible to call all evolution of development heterochrony when the term is not more strictly applied. However, classic examples of heterochrony that stand a more rigorous test may be present in the literature (see below). The question may thus become how to investigate putative cases using current methods. While flowering time per se may appear to be a phenotype straightforwardly interpreted as heterochronic, as we argue here, investigating whether a heterochronic gene pathway is affected would help to direct conclusions on heterochronic evolution (Li and Johnston, 2000).

We propose that cases of heterochrony could be confirmed by three criteria. First, a member of a “heterochronic pathway” is affected. Second, this results in what can be interpreted as a heterochronic shift in the evolution of development. Third, phylogenetic evidence is available for the heterochronic shift to have occurred in a meaningful window of evolutionary time. This will usually mean that an ancestral character state can be inferred or is available, as is wild-type in the case of mutants. Such a more narrow definition brings along clearer ways to test cases of heterochronic evolution which is otherwise not possible. Cases of heterochronic evolution or heterochronic mutations can be investigated by monitoring members of the known heterochronic pathway in development. If their action has modified in comparison to the ancestral form or in comparison to the wild-type, this can be taken as evidence for heterochrony. As is often the case in evolution and development, it is difficult to pinpoint exactly which gene has been affected in evolution. An initial approach to this problem is to investigate whether one of the microRNA’s or one of their targets show a changed expression pattern in development. More rigorously, the candidate gene can be further characterized using transgenic or genetic approaches. Thoroughly investigating heterochronic evolution at the molecular level should be helpful in correctly identifying the type of heterochrony and determining whether onset, rate or offset of developmental time has changed. In addition, the relative frequency of this heterochrony versus other modes of developmental evolution can be investigated and the types of morphological consequences can be described.

## TWO HETEROCHRONIC MUTANTS: *Antirrhinum incomposita* AND *choripetala*

**FIGURE 4:**
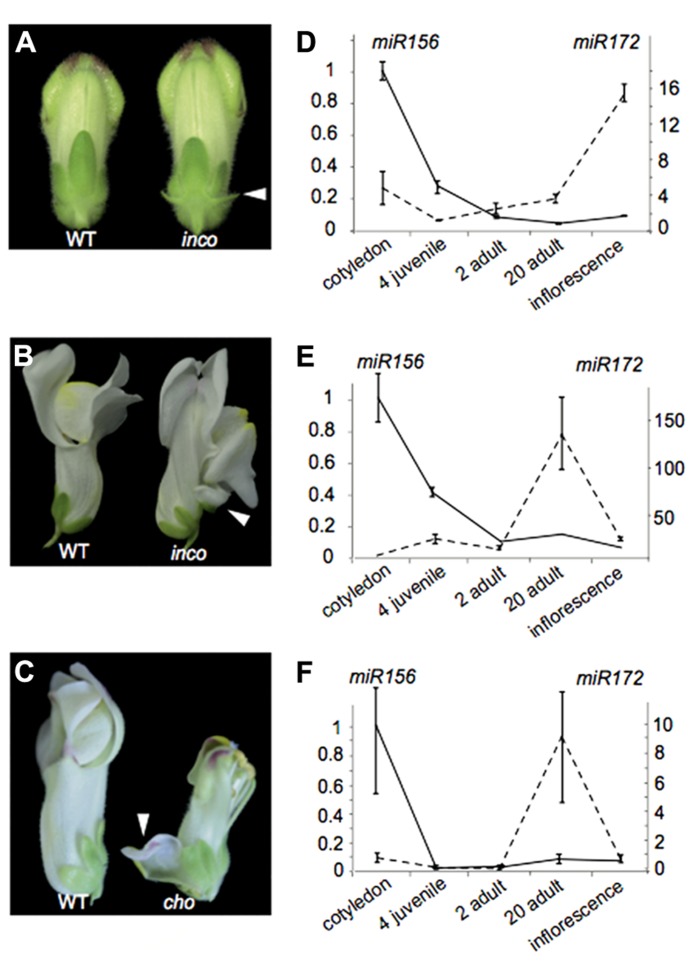
**The heterochronic *Antirrhinum* mutant *choripetala*.(A)** In *incomposita*, prophylls develop which are absent in wild-type *Antirrhinum* flowers. **(B)** *Inco* also occasionally develops petaloid sepals. **(C)** Petaloid sepals and an unfused corolla can also be observed in *choripetala*. **(D)** The expression pattern of *miR156* (left axis) and *miR172* (right axis) in *Antirrhinum* is similar to what has been observed for wild-type *Arabidopsis* and other species. Both in *inco ***(E)** and in *cho ***(F)**, an early increase in miR172 can be observed late in adult development and lower expression levels are present in inflorescence tissue. Error bars represent standard errors of three technical replicates. A second biological replicate gave similar results.

We illustrate these above points by investigating the *Antirrhinum majus* *incomposita *(*inco*) and *choripetala* (*cho*) mutants. The *inco* mutant has been characterized both morphologically and genetically in Masiero et al. (2004) and Wilkinson et al. (2000). While *inco* is affected in an ortholog of the *Arabidopsis* MADS-box gene *SVP*, the molecular basis of the *cho* phenotype remains unresolved. *Inco* flowers characteristically develop prophylls or bract-like structures at the base of their flowers, while these are absent from wild-type *Antirrhinum* flowers (**Figure 4A**). Some flowers also display a petaloid sepal phenotype, which *inco* has in common with *cho* (**Figures 4B, C**). The petaloid sepal phenotype in *inco *is strongly enhanced in a *cho* background, suggesting that *INCO *and *CHO* are functionally related in controlling sepal identity. At least for *cho* it has been shown that the petaloid sepals show ectopic B-class gene expression (Wilkinson et al., 2000).

From a morphological point of view, the ontogeny of *inco* and *cho* mutants has been compared to wild-type flower development using scanning electron microscopy. Such an analysis has the potential to reveal shifts in the relative timing of organ development, or heterochronies. Indeed for *inco*, the initiation of the lateral sepals is delayed and the primordia are displaced toward the center of the flower primordium. Probably as a consequence, the lateral sepals become fused and petaloid in *inco *(Masiero et al., 2004). Comparative ontogeny of wild-type and cho revealed that the sepals are narrower at initiation and their growth rate is reduced. This also makes *cho* interesting from a heterochronic point of view (Wilkinson et al., 2000).

To understand whether the heterochronic morphologies of *inco* and *cho* can be explained by modifications to the regulation of heterochronic pathway genes, we investigated in both wild-type and *cho* biological replicates the expression patterns of mature microRNA’s 156 and 172 using stem-loop qRT-PCR relative to the housekeeping gene actin. While we retrieve the expected expression pattern in wild-type plants (**Figure 4D**), remarkably in *cho* and more strongly in *inco* *miR172* expression is notably higher late in adult development only to strongly decrease rather than increase when flowers develop (**Figures 4E, F**). These expression patterns illustrate that modifications in developmental timing can be complex. It would also be difficult to classify *cho* as pre-displaced or neotenic because of the combination of rate effects. Furthermore, if the phenotypes can be (partially) explained by this changed expression pattern, our observations contribute to the notion that heterochronic phenotypes can be diverse.

## PUTATIVE CASES OF HETEROCHRONIC EVOLUTION IN PLANTS

While in the previous paragraphs we provided a heterochronic interpretation of mutant phenotypes, a number of putative classic cases of heterochronic evolution can be investigated now that a heterochronic pathway has been elucidated. The question is whether the pathway is affected in these instances and how?

A series of studies in evolution and development has tried to correlate expression of B-function genes to petal identity in sepals or bracts. These studies inconsistently did, or did not observe ectopic expression of B-function (Litt and Kramer, 2010; Ronse De Craene and Brockington, 2013). It will be interesting to re-investigate some of these studies in light of the idea that *APETALA2* is able to repress floral homeotic functions from the outer whorls as shown in *Arabidopsis* (Krogan et al., 2012). In the two cases in core eudicots we investigated, *Davidia involucrata* and *Impatiens hawkeri*, there was no clear indication of maintained heterotopic B-function expression (Geuten et al., 2006; Vekemans et al., 2012). If the causative gene indeed appears to be *APETALA2*, most of this type of morphological diversity may be heterochronic in nature rather than heterotopic as was previously proposed (Albert et al., 1998).

Heterotopic expression of showy characters outside the flower usually is also accompanied with modifications in organ shape and size. In some of these cases, ectopic expression of SVP has been implicated. The original observations derive from the maize *Tunicate* mutation (Han et al., 2012; Wingen et al., 2012). One of the best characterized naturally occurring cases of this modification in evolution is the inflated calyx syndrome in *Physalis *for which it was demonstrated that a homolog of *SVP *is heterotopically (or heterochronically?) expressed (He and Saedler, 2005). A link with either the microRNA’s or *APETALA2* expression has not been made thus far and awaits investigation. Yet such a link would not be too surprising as SVP is known to regulate *miR172* in *Arabidopsis* (Cho et al., 2012a).

The occurrence of cleistogamy has also been considered as heterochronic in the sense that the cleistogamous flowers relative to their chasmogamous flowers appear to end development prematurely (Lord and Hill, 1987). An extensively studied example is the one of *V. odorata* (Mayers and Lord, 1983a, b). This violet develops cleistogamous flowers in response to long days and chasmogamous flowers in response to short days. Similar to the effect in flowers is the change in shoot identity in relation to changes in photoperiod that can be observed in the leaves. Small cleistogamous flowers develop in the axil of leaves with long petioles, while chasmogamous flowers develop in the axil of small leaves with short petioles. *V. odorata* thus displays heterochronic variation in both flower and leaf development.

An example from gymnosperms is the spruce *acrocona* mutant, which develops reproductive cones with vegetative characters (Carlsbecker et al., 2013; Ruelens and Geuten, 2013). A final classic example of heterochronic variation in leaf shape can be found in Marsilean ferns (Allsopp, 1967; Pryer and Hearn, 2009). The leaves of the genus *Marsilea *develop different leaf shapes depending on environmental conditions. One condition is submergence under water. These types of leaves are also present in the evolutionary related genera, *Pillularia* and *Regnellidium. *Even more reduced leaves can be found in the related genera *Azolla* and *Salvinia*. Molecular data on these last two classic examples of heterochrony is not available yet, but could clarify if and how the heterochronic pathway can evolve.

## THE IMPORTANCE AND APPLICABILITY OF HETEROCHRONY

Heterochrony in animal evolution and development has been recognized as the major evolutionary mechanism contributing to diversity (De Beer, 1951; Gould, 1977). In comparison to animals, the role attributed to heterochrony in the evolution of plant development is historically smaller, not necessarily reflecting the biological significance of the concept. Indeed, notable exceptions, such as Armen Takhtajan, acknowledged a major role for heterochrony in plant evolution. Heterochrony by these proponents has been used to explain major, still outstanding questions in botany, such as the neotenic origin of the flower (Takhtajan, 1976). We propose that the molecular study of putative cases of heterochrony will assist in assessing the relative frequency of this type of developmental evolution in comparison to other types.

There are obvious and previously noted methodological difficulties when applying the idea of heterochrony to plants. The open development of plants was originally thought to be more difficult to study from a heterochrony point of view (De Beer, 1951). As plant development initiates in closed buds, the primordia are not easily visualized in a dynamic manner. Therefore, measuring the rate of development usually is indirect, through the use of an average developmental rate over a prolonged period of time. For instance the counting of leaves that developed in a certain time. For flower development, most studies lack data on either growth rate or relative timing of events. Average developmental rate cannot be measured and needs to be estimated from comparative floral ontogenetic work. While difficult, several of these examples have been reported though not necessarily recognized as heterochrony, such as the *incomposita* and *choripetala* mutants described above (Wilkinson et al., 2000; Masiero et al., 2004). A reason why heterochrony in leaf development (heteroblasty) is thought to be difficult to study is that many plants lack clear morphological markers for the transition from the juvenile to the adult and sexually mature stage. However, now that clear molecular markers are available to study the transition from the juvenile to adult phases in development, this difficulty can be overcome.

We would argue that plants, because they retain previous developmental stages in the adult form, are excellent models to study heterochrony. Even now, our current thinking about plant morphology could be named “heterochronic.” For example the idea of a carpel as essentially a folded young leaf reveals this (Arber, 1937; Takhtajan, 1976).

## MATERIALS AND METHODS

*C-fern *(*Ceratopteris*)* spores* were obtained from Carolina Biologicals (NC, USA). *Antirrhinum majus* *choripetala* and wild-type seeds were obtained from IPTK Gatersleben. For *Ceratopteris richardii*, spores were germinated in liquid (for gametophytes) or on solid (for sporophytes) Basic C-fern medium (NC, USA) in a Conviron Adaptis growth cabinet at 25°C under 200 μmol photons per meter squared per second (photosynthetic photon flux density PPFD) of cool white light in a 16 h light, 8 h dark daily cycle. To sample gametophytes, spores were germinated in an erlenmeyer and harvested 10 days after inoculation by centrifugation. Sporophytes were germinated in Magenta jars and sampled in triplicates 30, 44, 112, 119, 126, 133, and 144 days after plating on solid medium. *Antirrhinum* wild-type and *incomposita *seeds were germinated in the same light conditions as *Ceratopteris *in Magenta jars on 1/2; Murashige and Skoog medium. Seedlings were transferred to soil and placed in a growth chamber also at 25°C with approximately 100 PPFD of cool white light. After homogenization in liquid nitrogen, RNA was extracted using the Plant RNA Reagent (Invitrogen) for *Ceratopteris* and using TRIzol (Invitrogen) for *Antirrhinum* according to the manufacturers protocol. DNA present in the RNA prep was degraded using Turbo DNase (Inivitrogen) and degradation was confirmed by PCR amplification of actin and evaluation using agarose gel electrophoresis. DNA free RNA was then reverse transcribed using the cDNA Reverse Transcription kit (Promega) according to the manufacturers procedures. Before using the cDNA for qRT-PCR, whether the cDNA was amplifiable was tested using regular PCR amplification of actin.

Expression analysis in *Ceratopteris *was performed using qRT-PCR and normalized relative to actin. Primers for APETALA2 expression were 5′-CAGCATCATCAGGATTCTCACATAT-3′ and 5′-GACATGGTAGATGCGGAGCTAGT-3′ and for actin 5′-TCC-TCGGGCTGTATTTCCTAGTAT-3′ and 5′-CCTCATCACCAAC-ATATGCATCTT-3′. For *Antirrhinum, *cDNA was prepared using a combination of stem-loop and oligo-dT primers. Stem-loop primers were 5′-GTCGTATCCAGTGCAGGGTCCGAGGTATTC-GCACTGGATACGACGTGCTC-3′ and 5′-GTCGTATCCAGTGCAGGGTCCGAGGTATTCGCACTGGATACGACATGCAC-3′ to amplify the mature microRNA’s.

## Conflict of Interest Statement

The authors declare that the research was conducted in the absence of any commercial or financial relationships that could be construed as a potential conflict of interest.

## Contributions

Koen Geuten conceived the ideas in the manuscript. Koen Geuten and Heleen Coenen wrote the manuscript. Experiments were performed through technical assistance.
